# Cardiovascular risk prevention in children

**DOI:** 10.1186/1824-7288-40-S1-A20

**Published:** 2014-08-11

**Authors:** Francesco Martino

**Affiliations:** 1 Department of Pediatrics and Child Neuropsychiatry, Sapienza University of Rome, Italy

## 

Cardiovascular disease (CVD) is the major cause of mortality in industrialized countries. Atherosclerosis is characterized by a long lag time between onset and clinical manifestations. There is evidence that CVD initiate before birth by developmental changes in utero. Genetic and environmental factors may interact in specific periods of life (prenatal, perinatal, early childhood), giving rise to altered developmental plasticity and epigenetic modifications with abnormal phenotypic expression of genetic information without altering DNA sequence. Nutritional imbalances and other environmental clues may cause intrauterine growth delay, decreased gestational age, low birth weight and postnatal catch-up growth. Fetal exposure to maternal hypercholesterolemia has been associated with increased risk and progression of atherosclerosis. Oxidative stress seems to play a major role in the atherosclerotic process by activating both platelets and monocytes forming proinflammatory and proaterogenic substances, resulting in endothelial dysfunction, decreased flow-mediated dilation, and increased carotid intima-media thickness [1,2]. CVD primary prevention should be implemented early. High risk, apparently healthy [3] subjects should be identified , and treated early by healthy eating, physical activity encouragement, healthy lifestyle, the possible addition of nutraceuticals [4] and adding drugs if risk profile is not decreased. Restoration of endothelial function in the reversible phase of atherosclerosis is an essential step. Epigenetics might provide novel and early markers to identify those at risk, thereby developing innovative therapies.
